# Cerium Oxide Nanoparticles Reduce Microglial Activation and Neurodegenerative Events in Light Damaged Retina

**DOI:** 10.1371/journal.pone.0140387

**Published:** 2015-10-15

**Authors:** Lavinia Fiorani, Maurizio Passacantando, Sandro Santucci, Stefano Di Marco, Silvia Bisti, Rita Maccarone

**Affiliations:** 1 Department of Applied Clinical Science and Biotechnology, University of L'Aquila, Via Vetoio, Coppito II, 67100 L'Aquila, Italy; 2 Department of Physical and Chemical Sciences, University of L'Aquila, Via Vetoio, Coppito I, 67100 L'Aquila, Italy; University of Cologne, GERMANY

## Abstract

The first target of any therapy for retinal neurodegeneration is to slow down the progression of the disease and to maintain visual function. Cerium oxide or ceria nanoparticles reduce oxidative stress, which is known to play a pivotal role in neurodegeneration. Our aim was to investigate whether cerium oxide nanoparticles were able to mitigate neurodegeneration including microglial activation and related inflammatory processes induced by exposure to high intensity light. Cerium oxide nanoparticles were injected intravitreally or intraveinously in albino Sprague-Dawley rats three weeks before exposing them to light damage of 1000 lux for 24 h. Electroretinographic recordings were performed a week after light damage. The progression of retinal degeneration was evaluated by measuring outer nuclear layer thickness and TUNEL staining to quantify photoreceptors death. Immunohistochemical analysis was used to evaluate retinal stress, neuroinflammatory cytokines and microglial activation. Only intravitreally injected ceria nanoparticles were detected at the level of photoreceptor outer segments 3 weeks after the light damage and electoretinographic recordings showed that ceria nanoparticles maintained visual response. Moreover, this treatment reduced neuronal death and “hot spot” extension preserving the outer nuclear layer morphology. It is noteworthy that in this work we demonstrated, for the first time, the ability of ceria nanoparticles to reduce microglial activation and their migration toward outer nuclear layer. All these evidences support ceria nanoparticles as a powerful therapeutic agent in retinal neurodegenerative processes.

## Introduction

Retinal diseases like Retinitis Pigmentosa, glaucoma, Stargardt and Age related Macular degeneration (AMD) lead to a cascade of neurodegenerative events that share the common outcome of neuronal death and visual impairment see for ref. [1,2]. Even though many factors may contribute to the development and progression of different pathologies, the oxidative stress seems to play a relevant role in many of them including Stargardt and AMD [3,4]. Albino rats exposed to high intensity light are considered a good and reproducible model of oxidative stress [5]. A relatively short exposure to bright continuous light (BCL) induces localized and rapid changes in the outer retina. The initial focal damage leads to destabilization of photoreceptors in the adjacent retina, so that the damaged region increases in size, over time. The neurodegeneration starts in a dorsal area called “hot spot” [5,6]. The spatial coincidence of this area with peculiar morphological characteristics of photoreceptors and high density of ganglion cells leads to the notice of a homology of this area with the *area centralis* [5]. It was suggested that the focal damage in the visual centre of the albino rat retina is reminiscent of the histopathology of AMD [5]. Recently, microarray experiments showed that light exposure modulated the expression of many genes including neuroprotection-linked genes, oxidative stress linked genes, apoptotic-linked genes and inflammation linked genes [7]. As in human retinal diseases like AMD, also in light damaged rats the final outcome is photoreceptors death leading to progressive deterioration of visual function. The first target was to reduce the oxidative stress to maintain visual function as long as possible. To reach this goal the use of nanoparticles was a good strategy because they appear safe and effective [8]. Inorganic catalytic ceria or cerium oxide (CeO_2_) nanoparticles are antioxidants that possess regenerative radical scavenging activities due to the presence of oxygen vacancies on the surface of these nano-sized particles and to the auto-regenerative cycle between the two-oxidation states, Ce^3+^ and Ce^4+^. They have been successfully proposed as neuroprotectants and drug delivery devices see for ref. [1,9].

Although oxidative stress is essential to start the cascade of events leading to neurodegeneration, its progression can be attributed to the activation of many factors including release of chemokines and neuroinflammation [2]. Recently great attention has been addressed to the role of microglia see for ref. [5,10,11]. The so-called microglial dysfunction hypothesis articulates that the age-related susceptibility to neurodegenerative disease in human is causally connected to the age-related microglial deficiency in neuroprotective function. Microglia perform an active cross-talk with Müller cells to maintain constant levels of neurotrophic factors important for retinal physiology and monitor synaptic activities. In response to negative events including oxidative stress, microglia activate and can be responsible for the progression of degeneration in light damaged rats as well as in many retinal diseases [5,10,11]. We tested the possibility to reduce microglial activation by using CeO_2_ nanoparticles. Confirming previous results [8], one single intravitreal injection of CeO_2_ nanoparticles was able to protect retinal function in albino rats even though the light damage was induced three weeks after the injection. CeO_2_ nanoparticles remained active, localized in the retina and effectively slowed down the neurodegenerative process together with microglial activation. In addition, we tested intravenous injection, but this treatment was unable to protect photoreceptors.

## Methods

All experiments were conducted in accordance with ARVO statement for the use of animals in ophthalmic and vision research, authorization number 83/96-A of 29/11/1996, by the Ministry of Health and approved by local Ethical Committee of University of L’Aquila. Animals were born and raised in dim cyclic light conditions (12 hours light, 12 hours dark) with an ambient light level of approximately 5 lux.

Experiments were performed in n = 36 adult Sprague Dawley (SD) rats divided as described below:

1° Group (Control): n = 6 without any treatments.
2° Group (LD): n = 6 with light damage.
3° Group intravitreally injected: n = 6 with CeO_2_ nanoparticles in right eye (CeO_2_ NP) and 0.9% NaCl in left eye (Saline) before BCL.
4° Group (Vein): n = 6 with CeO_2_ nanoparticles injected through the tail vein before BCL.
5° Group FITC-CeO_2_ nanoparticles: with CeO_2_ nanoparticles labelled with fluoresceine isothiocianate (FITC) injected intravitreally (n = 6) and in the tail vein (n = 6).

All the injections and recordings were performed under Ketamine/Xylazine (10mg/100g – 1.2mg/100g) anaesthesia.

### Light Exposure

Animals were placed in plexiglass cages individually with food available on the floor and water in plastic bottles, dark-adapted overnight and at 9 a.m. exposed to 1000 lux for 24 hours. Immediately after, they were returned to dim cyclic conditions for one week.

### Intravitreal Injection of CeO_2_ Nanoparticles

Three weeks before BCL, the third group received 1 mM CeO_2_ nanoparticles in 0.9% NaCl (2 μl) (CeO_2_ NP) and 2 μl of 0.9% NaCl (Saline) were intravitreally injected using an Hamilton syringe.

### Injection Through The Tail Vein of CeO_2_ Nanoparticles

Three weeks before BCL, the fourth group (Vein) was injected through the tail vein with 300 μl of a suspension of CeO_2_ nanoparticles at the dose of 20 mg/kg.

### Electrophysiological Recordings

To evaluate visual function, flash electroretinogram (fERG) was recorded in different groups: LD, CeO_2_ NP/Saline and Vein, after one week from BCL.

fERG was recorded in dark-adapted retina in response to light-flashes of increasing intensity. The rats, anaesthetized by an intraperitoneal injection of Ketamine/Xylazine (10mg/100g-1.2mg/100g), were mounted in a stereotaxic apparatus, body temperature was maintained at 37.5°C, and heart rate was monitored. Corneas were anaesthetized with a drop of novocaine, and pupils were dilated with 1.0% atropine sulphate (Allergan, Westport, IR). Recordings were made from the eye, with a gold electrode loop (diameter = 2.0 mm) placed on the cornea while the contralateral eye was fully covered with a bandage. The reference electrode was placed on the contralateral cornea under the bandage, and the ground electrode was inserted in the anterior scalp, between the eyes. The rat’s head was positioned just inside the opening of the Ganzfeld dome (Biomedica Mangoni, Pisa, Italy). This electronic flash unit generated increasing stimulus in a 0.001–100 cd/m^2^ range. Responses were recorded to be over 300 ms plus 25 ms of pre-trial baseline, amplified differentially, bandpass filtered at 0.3 to 3 Hz, digitized at 0.25- to 0.3-ms intervals by a personal computer interface (LabVIEW 8.2; National Instruments, Milan, Italy), and stored on a disc for processing.

### Immunohistochemistry and Morphology

At the end of recording session, the eyes were enucleated and fixed in 4% paraformaldehyde for 6 hours, washed in 0.1 M phosphate-buffered saline (PBS, pH 7.4), and cryoprotected by immersion in 10%, 20% and 30% sucrose overnight. Eyes were embedded in the compound optimum cutting temperature (OCT) (Tissue-Tek; Qiagen, Valencia, CA), snap frozen in liquid nitrogen/isopentane, and cryosectioned at 20 μm. Sections were collected on gelatine and poly-l-lysine-coated slides and stained with propidium iodide to measure the thickness of outer nuclear layer (ONL). ONL was measured starting at the dorsal edge along the vertical meridian crossing the optic nerve head following a procedure already described [12]. Measurements are expressed as ratio ONL/total retina thickness and calculated for the entire retinal section. Cryosections were also immunolabelled for fibroblast growth factor (FGF2) protein (Upstate Biotechnology, Lake Placid, NY) Ionized calcium binding adaptor molecule 1 (IBA-1) (microglial marker, Wako Pure Chemical Industries, Japan) and Tumor Necrosis Factor α (TNF-α) (Santa Cruz Biotechnology, Inc.). To block nonspecific binding, 1% bovine serum (BSA) for FGF2 and 10% goat serum for IBA1 and TNF-α were used. Sections were incubated overnight at 4°C with mouse monoclonal FGF2 antibody diluted (1:200 in 1% BSA), rabbit polyclonal IBA1 (1:1000 in 1% goat serum) and Armenian hamster monoclonal TNF-α (1:200 in 1% goat serum). Secondary antibodies for FGF2 and IBA1 were anti-mouse and anti-rabbit IgG conjugated to green fluorescent dye (Alexa Fluor 488; Molecular Probes, Invitrogen, Carlsbad, CA) diluted at 1:200 and incubated at 37°C for 2 hours. For TNF-α biotin goat anti-hamster IgG and Streptavidin Cy3 were used.

Dying neurons were visualized using terminal deoxynucleotidyltransferase d-UTP nick end labelling technique (TUNEL) as previously described [13].

### Intravitreal and Intravenous Injection of FITC-CeO_2_ Nanoparticles

To visualize the CeO_2_ nanoparticles localization in the retina, 20 mg/kg of FITC-CeO_2_ nanoparticles [9] were injected in 12 SD rats: intravitreally (n = 6) and trough the tail vein (n = 6). The animals were euthanized at 24 h (n = 3+3) and 3 weeks after treatment (n = 3+3). The cryosections were processed for nuclear staining Bisbenzimide (Hoechst) to obtain visualization of FITC-CeO_2_ nanoparticles.

### Confocal Images

All the images reported were acquired by confocal microscope (Nikon 80i, Tokio, Japan). For comparison purposes, all images were taken using the same acquisition parameters.

### Images analysis

The study was carried out on retinal sections (from dorsal to ventral) which included the optic disc. The analysis were performed in six animals for each experimental group, three sections were analysed for each eye.

Mean fluorescence intensity analysis for FGF2 was performed using ImageJ software.

### Statistical Analysis

Statistical analysis was performed by Student’s t-test and one-way ANOVA test followed by Tukey test.

## Results

### Synthesis, Characterization and Retinal Distribution of Cerium Oxide Nanoparticles

CeO_2_ nanoparticles were synthesized using a modified precipitation method with cerium nitrate hexahydrate as the precursor [14]. The detailed procedure of synthesis can be found in a previous work [15]. In particular CeO_2_ nanoparticles used in this work, were calcined in an air furnace at temperature T = 500°C for 8 hours. The obtained sample was studied by means of X-ray diffraction (XRD), (SIEMENS D5000 diffract meter with Cu *K*_α_ operating in the Bragg–Brentano mode) and X-ray photoemission spectroscopy (XPS) (PHI 1257 ESCA system, base pressure of 1×10^−9^ Torr, equipped with a Mg *K*_α_ photon source and a hemispherical analyser) techniques. From these analyses, we determined the lattice parameter and crystallite size, and the relative amounts of Ce^4+^ and Ce^3+^ spices of the CeO_2_ nanoparticles.

**Fig 1:**
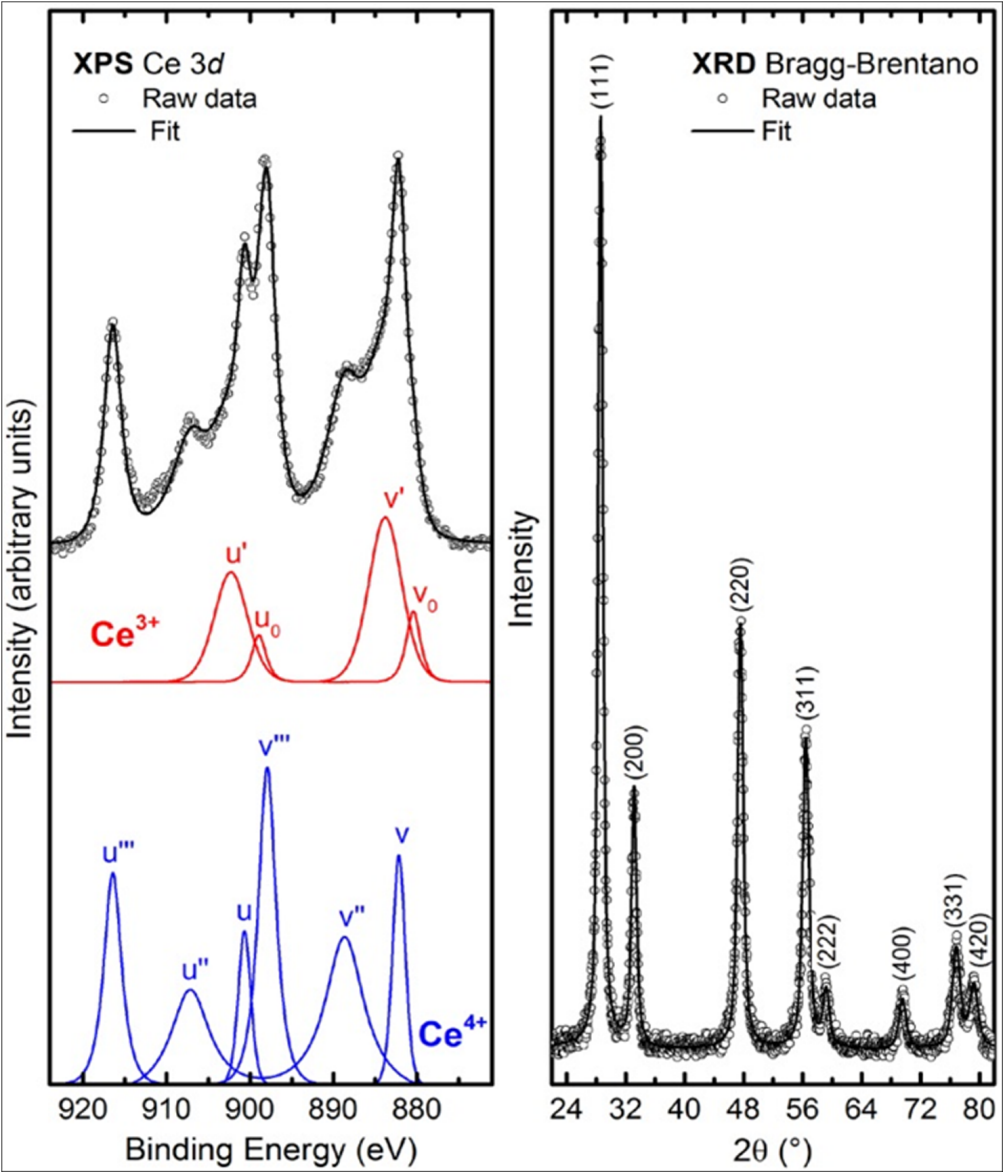
Electronic and structural properties of CeO_2_ Nanoparticles. Decomposition of the XPS Ce 3*d* core level into Ce^3+^ and Ce^4+^ emission (left panel) and XRD pattern (right panel) for the as prepared CeO_**2**_ nanoparticles.

XPS spectrum (Fig 1 left panel) showed the deconvoluted Ce 3*d* core-level spectra of the as-prepared CeO_2_ nanoparticles. The complicated structure of the spin-orbit split Ce 3*d*_5/2_ and Ce 3*d*_3/2_ core levels (located at about 884 and 902 eV, respectively) result from various initial and final states [15]. Specifically, v_0_, v', u_0_ and u' in Fig 1 are attributed to Ce^3+^ (red lines), whereas v, v", v‴, u, u" and u‴ are characteristic peaks of Ce^4+^ (blue lines). A semiquantitative analysis of the integrated peak area can provide the concentration of Ce^3+^ ions in the synthesized nanoparticles. It can be calculated as [15], and in our case, we obtained a percentage of 26.4%.

XRD pattern (Fig 1 right panel) shows that the CeO_2_ nanoparticles are single phase and correspond to cubic phase fluorite-type CeO_2_ (JCPDS 034–0394). From XRD fitting procedure, as reported [15], we have obtained a lattice parameter of 0.54155 nm and crystallite size of 14.96 nm. We noted that the lattice parameter is larger than that of the bulk CeO_2_, 0.54113 nm (JCPDS 034–0394). From these results and those reported in the literature [15], we confirmed that an increase in the lattice parameter was always accompanied by a change in the oxidation state of cerium, due to the lattice strain induced by the creation of oxygen vacancies and the introduction of Ce^3+^ ions in the cerium oxide crystal structure. Consequently, an increase of the Ce^3+^ was mainly localized on the surface of the nanoparticle. Therefore, the ability of CeO_2_ to change the oxidation state of Ce between Ce^3+^and Ce^4+^ played a critical role in its catalytic and medicinal functionalities.

In addition, in this study, we prepared CeO_2_ nanoparticles conjugated with FITC to analyse their bio distribution in the retina after intravitreal or intravenous injection by means of confocal microscopy. Therefore, CeO_2_ nanoparticles surface was functionalized through the addition of primary amines, using amino-propyl-trimethoxysilane (APTMS) in order to attach alkoxysilane groups to the surface hydroxyl groups. These primary amino groups are thus available for the attachment of dye FITC [9].

**Fig 2:**
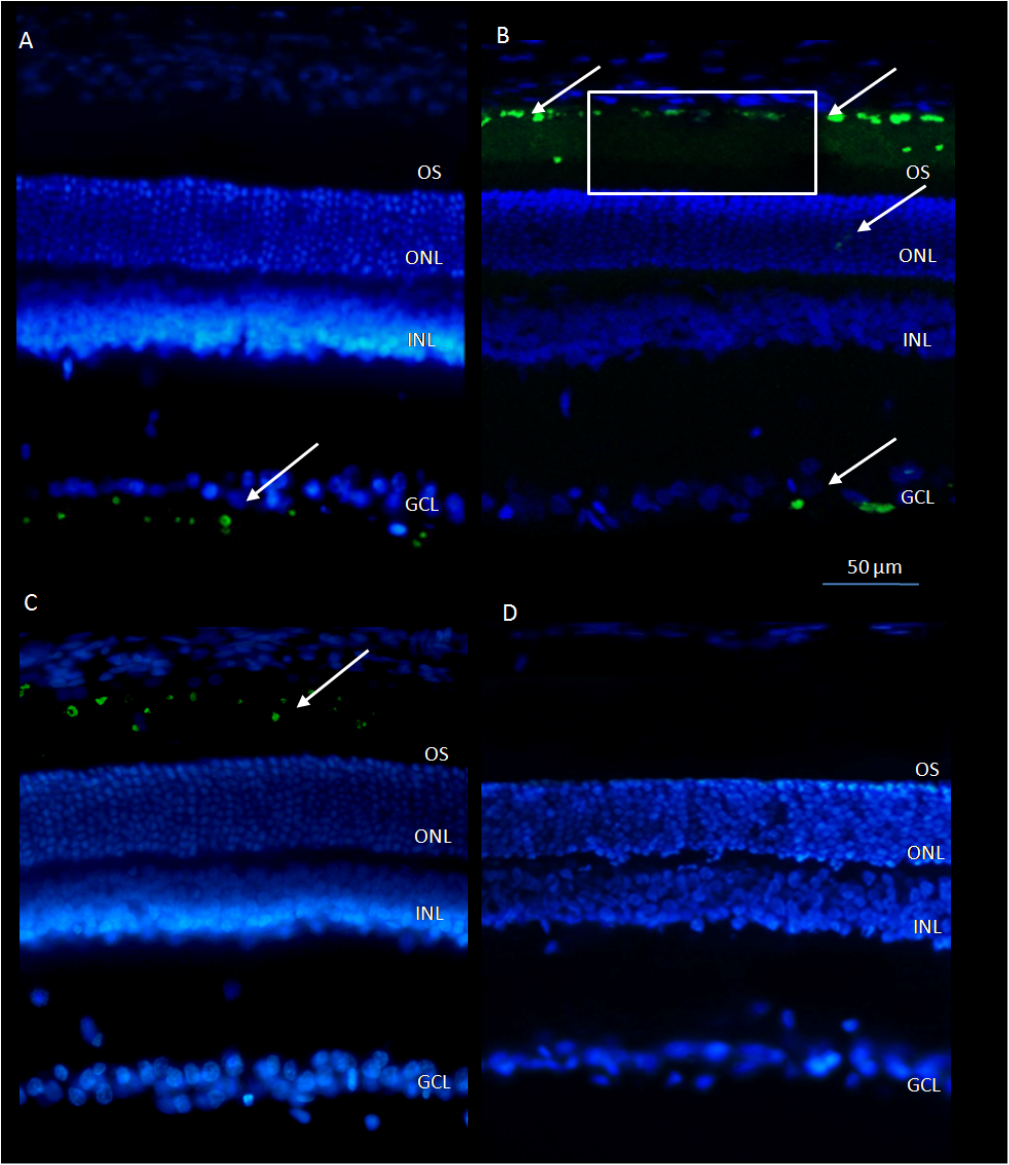
FITC-CeO_2_ nanoparticles in retinal sections labelled with bisbenzimide. Panels A-B: intravitreal injection of FITC-CeO_2_ NPs in rats euthanized after 24 h and after 3 weeks respectively. Panels C-D: injection trough the tail vein of FITC-CeO_2_ NPs in rat euthanized after 24 h and after 3 weeks respectively. Images have been acquired with confocal microscopy. In panel B the white rectangle represents the bleaching of FITC-CeO_2_ NPs. Scale bars: (A-B-C-D) 50μm. OS: outer segment, ONL: outer nuclear layer, INL: inner nuclear layer, GCL: ganglion cell layer.

FITC-CeO_2_ nanoparticles were injected intravitreally and intravenously and rats were euthanized after 24 h and after 3 weeks. FITC-CeO_2_ nanoparticles intravitreally injected after 24 h were localized at the level of ganglion cell layer (Fig 2A), after 3 weeks (Fig 2B) were detected, as indicated by the arrow, also in the ONL, few dots on the outer plexiform layer (OPL) and strongly in the outer segment (OS). After the intravenous injection, it was possible to localize FITC-CeO_2_ nanoparticles only after survival time of 24 h (Fig 2C), at level of photoreceptors OS. No fluorescent signal was detected after 3 weeks (Fig 2D). Only two-time points were checked. As already reported [9], it is possible to visualize labelled particles only when aggregated; accordingly, it is impossible to document their continuous distribution. In addition, white rectangle in Fig 2B shows that FITC-CeO_2_ nanoparticles are easily bleached after few seconds of laser exposure during confocal imaging.

### Retinal Function

**Fig 3:**
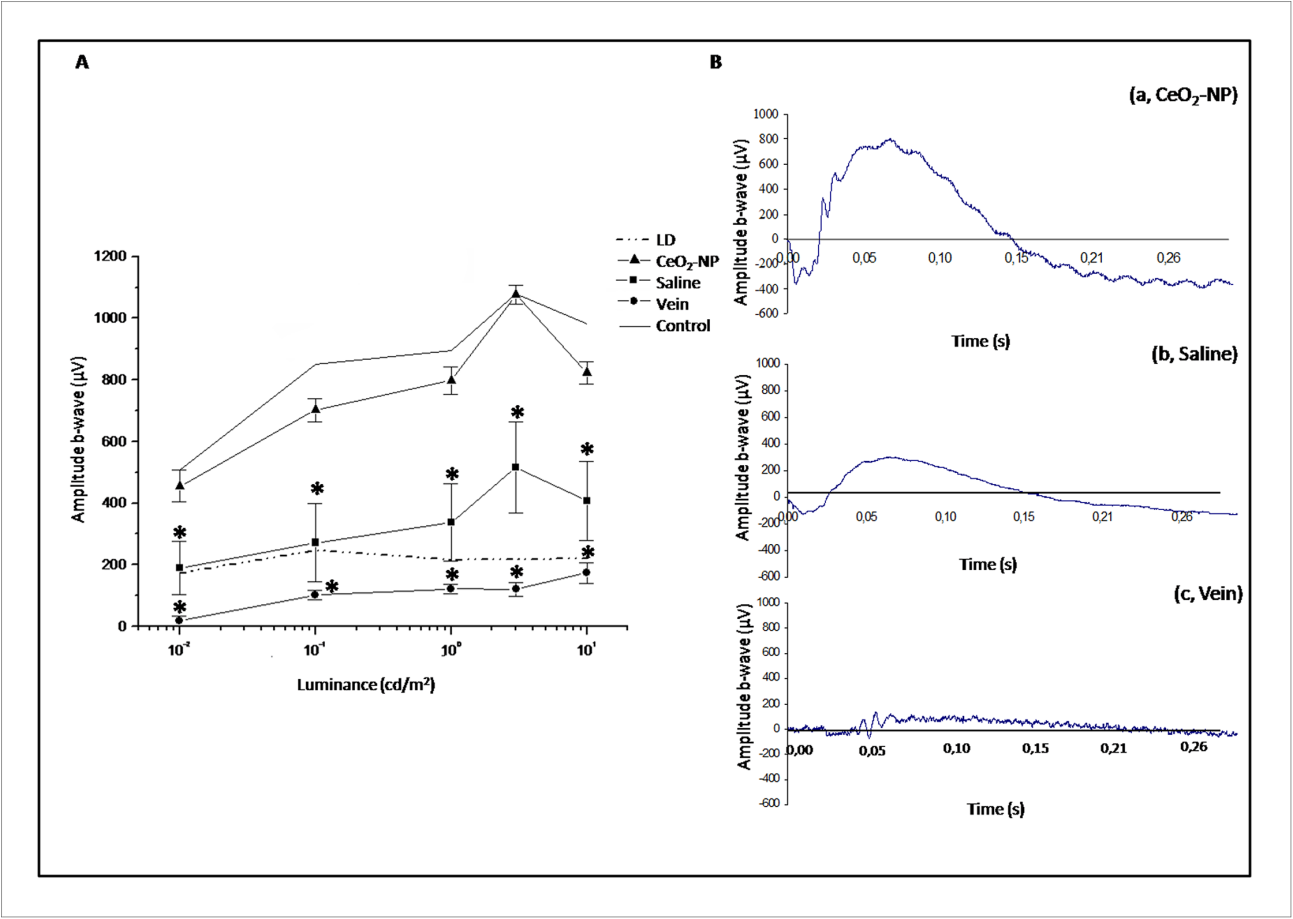
Functional analysis. Panel A. Mean b-wave fERG amplitude (μV) plotted against luminance (cd/m^2^). The mean ± SE is reported for each experimental group (n = 6 for each group). Dash dot-dot and continuous line represent the trend of LD Control group, respectively. Panel B: Representative fERG response in the three experimental conditions (CeO_2_ NP, Saline, Vein) at 3 cd/m^2^. Statistical analysis was performed, for each group versus CeO_2_ NP, using one-way ANOVA followed by Tukey test. *P< 0.05.

To verify the efficacy of the nanoparticles developed by our team, we referred to a standardized model already used [8]. Before exposing animals to BCL we recorded fERG from both eyes and no major changes were detected (data not shown), confirming previous observations [8]. One week after exposure to damaging light, we recorded fERG in dark-adapted conditions. The amplitude of b-wave as a function of stimulus intensity is reported in Fig 3 panel A. In agreement with previous data, the amplitude of the b-wave was strongly reduced after light damage [16]. Intravitreal treatment largely preserved retinal function, while fERG recorded from contralateral eye (Saline) and in Vein treated animals showed a strong reduction of the b-wave amplitude from threshold to saturation. Intravenous injection did not provide any protection to light induced retinal damage. The continuous and dash dot-dot line in Fig 3 panel A represent the average amplitude of b-wave in Control and LD groups, respectively. An example of recording at fix luminance (3 cd/m^2^) is reported in Fig 3 panel B.

### Retinal Morphology and “Hot Spot” Analysis

We analysed the entire retina, from dorsal to ventral crossing the optic nerve head, to evaluate the spatial extension of degeneration induced by BCL and to compare it among experimental conditions.

**Fig 4:**
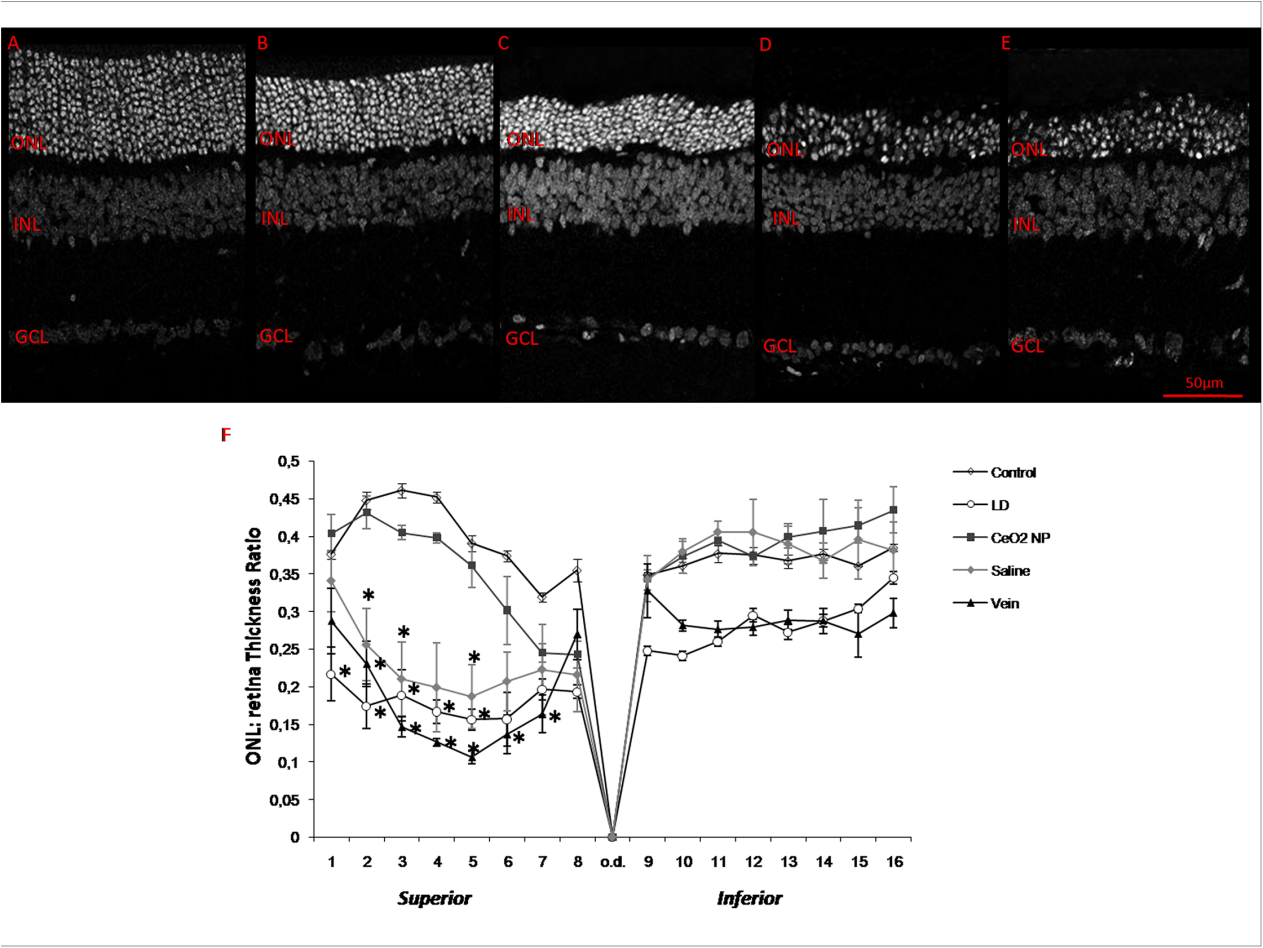
Thickness of ONL in all experimental conditions. Representative sections from superior retina (1 mm from optic disc) of Control (A), CeO_2_ NP: (B), Saline: (C), Vein: injected trough the tail vein (D) and LD (E). In all panels nuclei, labelled with propidium iodide, are reported in black and white. Panel F shows ONL thickness as a function of distance from the superior to the inferior edge crossing optic disc. Measurements are expressed as ratio ONL/total retina thickness. Statistical analysis was performed by one-way ANOVA followed by Tukey test for each group versus CeO_2_ NP group. Data are shown as mean ± SE; (n = 6 for each group). *P< 0.05, **P<0.01. ONL: outer nuclear layer, INL: inner nuclear layer, GCL: ganglion cell layer.

**Fig 5:**
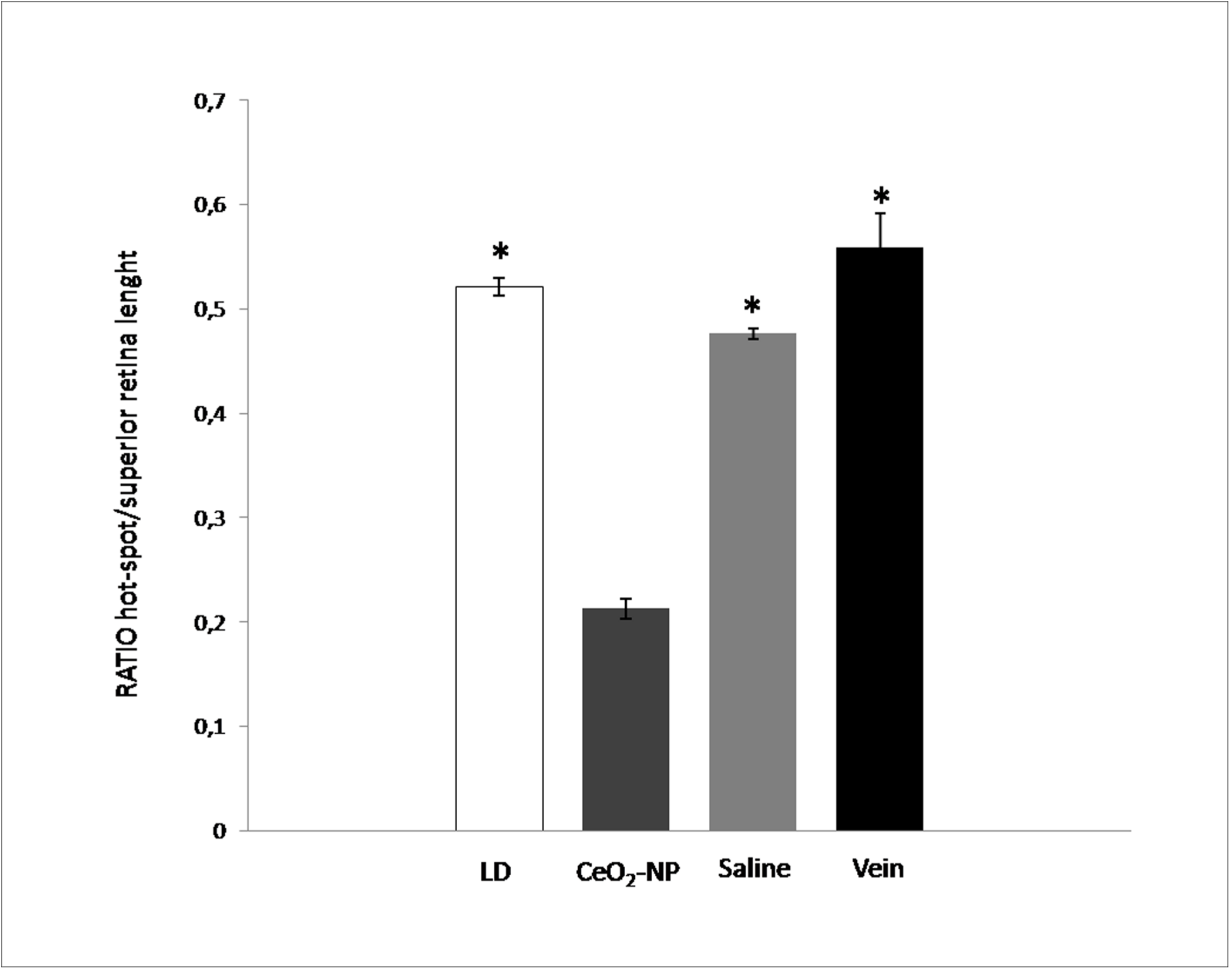
Analysis of “hot spot” region. The graph represents the ratio between “hot spot” and superior retina length in the four experimental conditions. Statistical analysis was performed, for each group versus CeO_2_ NP group, using one-way ANOVA followed by Tukey test (n = 6 for each experimental group). *P<0.05.

Fig 4 shows data obtained from the section stained with propidium iodide in all experimental groups. Images A (Control), B (CeO_2_ NP), C (Saline), D (Vein) and E (LD), captured in black and white mode, show the retinal region corresponding to the “hot spot”. It can be easily noted that the ONL of CeO_2_ NP (Fig 4B) appears better organised compared to Fig 4C, 4D and 4E suggesting that CeO_2_ nanoparticles, injected into the vitreous, are able to modulate the damage induced by BCL. To better assess the rate of photoreceptors survival, the measured thickness of the ONL across to the entire retina from dorsal to ventral, crossing the optic disc are reported in Fig 4F. As already observed, BCL induces maximal thinning in the ONL in a dorsal position, named “hot spot”. The protective effect of CeO_2_ nanoparticles, injected intravitreally, was particularly evident in that area, where together with the maintenance in ONL thickness there was also a reduction in the total extension of the “hot spot”. We measured its extension and we reported in Fig 5 the results obtained from the four experimental conditions, normalised with respect to dorsal retinal extension. The difference in length between CeO_2_ NP and the other three experimental groups was statistically significant.

### Cell Death and Microglial Activation

**Fig 6:**
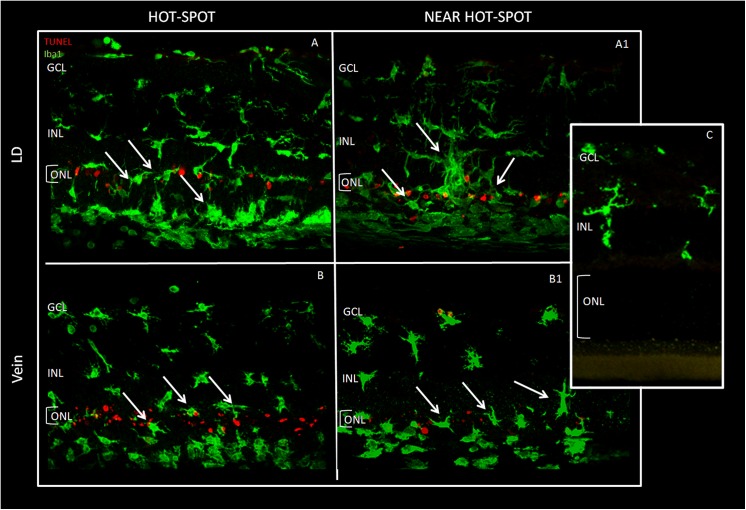
Microglia and TUNEL images in LD and intravenous injection. Immunolabelling for microglia (anti-Iba1) in green and apoptotic nuclei in red. The figure shows two different parts of superior retina, in each experimental group, one week after BCL. The arrows indicate the presence of activated microglia in the ONL. Panels A-B: “hot spot” region in LD and Vein; panels A1-B1: “near hot spot” in LD and Vein; panel C: control. ONL: outer nuclear layer, INL: inner nuclear layer, GCL: ganglion cell layer.

**Fig 7:**
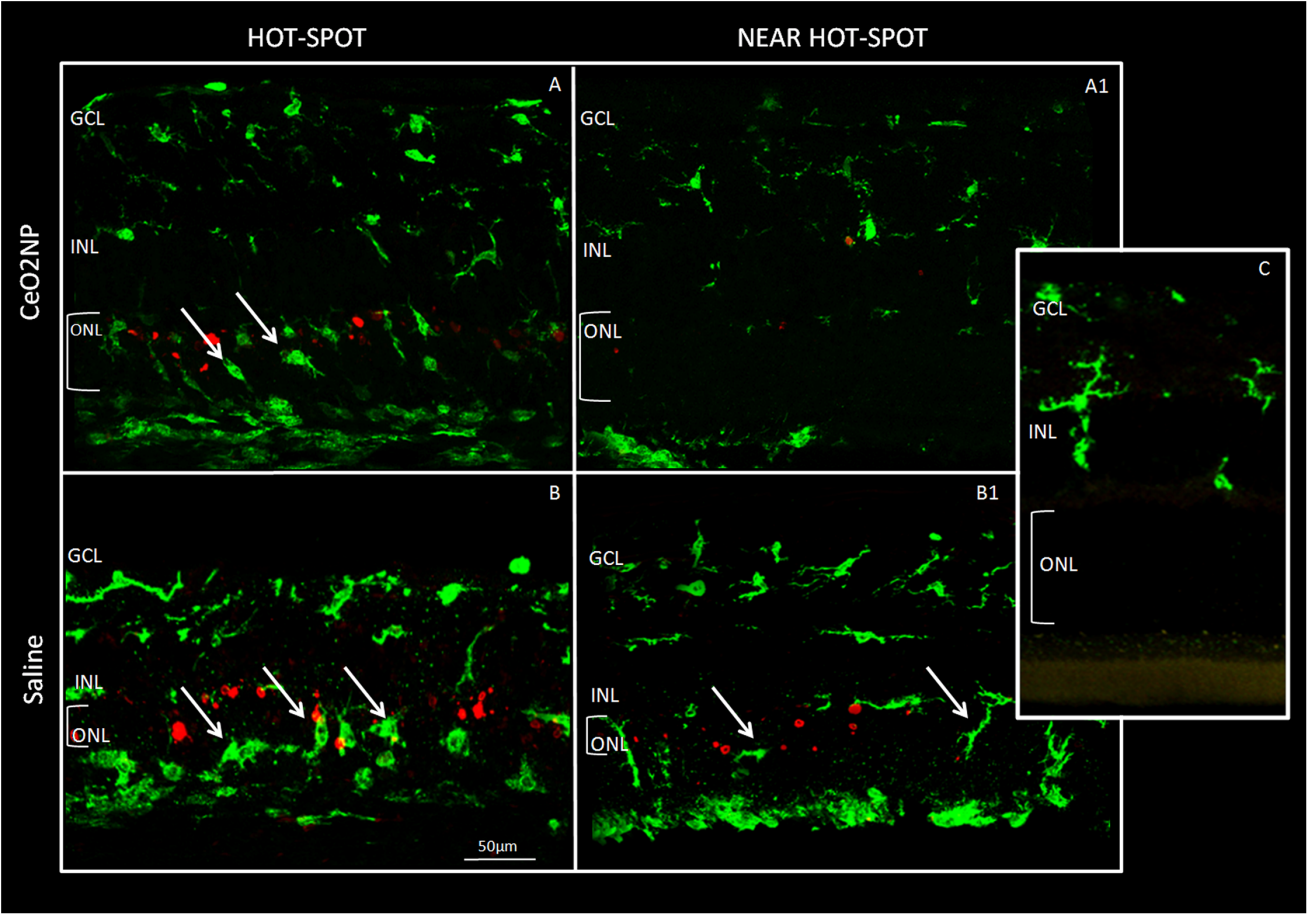
Microglia and TUNEL images in intravitreal injection. Panels A-B: “hot spot” region in CeO_2_ NP and Saline group; Panels A1-B1: “near hot spot” in CeO_2_ NP and Saline; panel C: Control. Abbreviations and arrows as in Fig 6.

**Fig 8:**
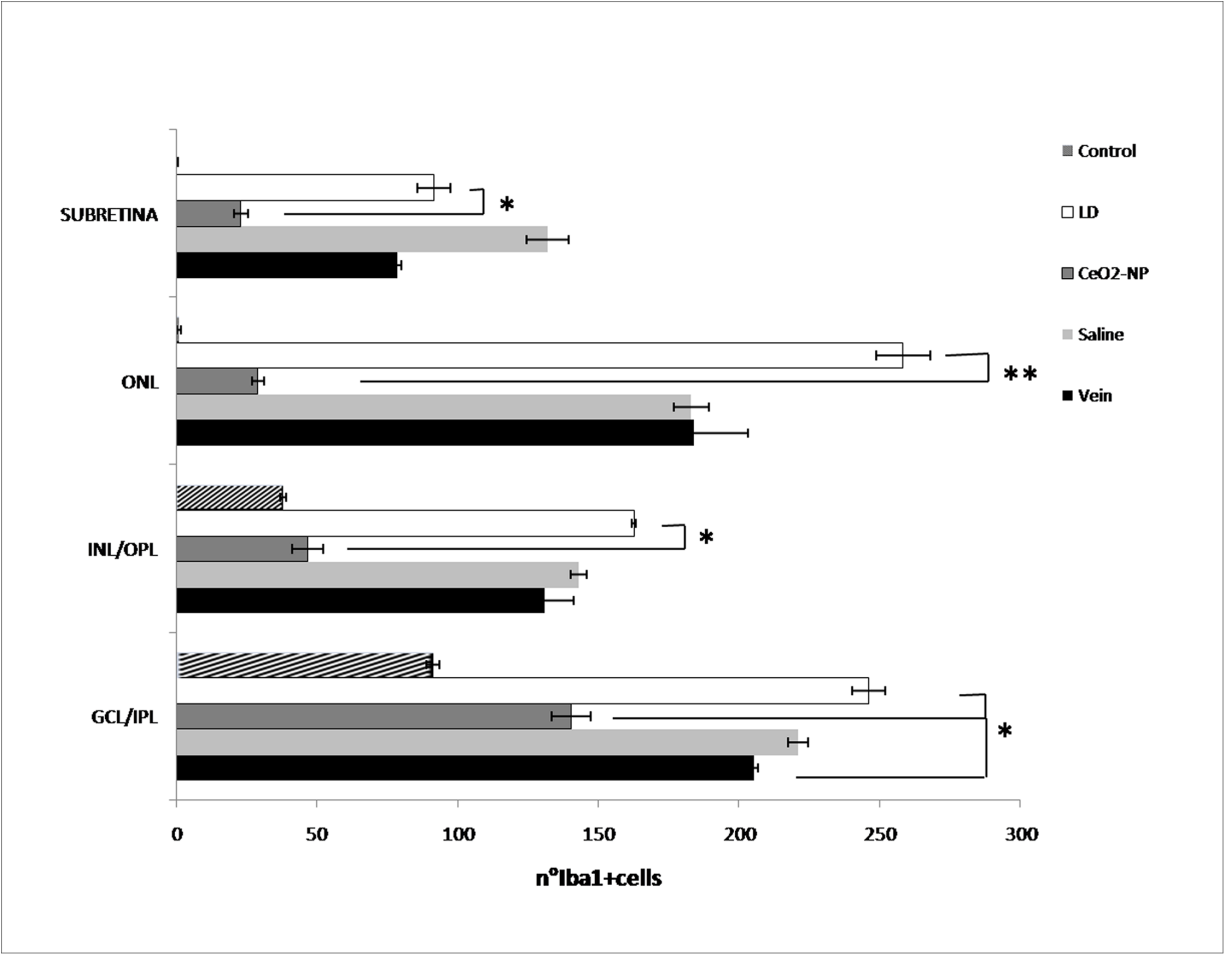
Microglia quantitative analysis. Comparison of Iba1-positive cells in each retinal layer in Control, LD, CeO_2_ NP, Saline and Vein groups. Ceria nanoparticles in CeO_2_ NP group induce a significant decrease of Iba1-positive cells mainly in ONL compared to LD group. The mean ± SE is reported. *P˂0.05, **P˂0.001 (Student’s t-test).

To further analyse relevant events induced by BCL such as neuronal death and inflammatory response, we labelled retinal sections with TUNEL technique to quantify apoptotic figures. To visualize the recruitment of microglia/monocytes we used Iba1 marker. In the Control group, microglia, in their inactive form, were only present in the inner retina. After BCL (LD group), Iba1-positive nuclei appeared in the ONL and particularly in the “hot spot” region, as it occurred for TUNEL-positive cells. Representative images of the five experimental groups, taken in corresponding dorsal retinal regions, are reported in Figs 6 and 7. In particular, for both figures, panels A and B represent the “hot spot” region, while panels A1 and B1 correspond to a region near the “hot spot”, panel C represents the Control. It is interesting to note that in the centre of the “hot spot”, for all tested groups, there was a great number of both TUNEL and Iba1 positive cells, while moving away from the centre of the “hot spot”, their number decreased only in the intravitreally treated eye group (CeO_2_ NP). A detailed analysis of Iba1 positive figures along the entire retina and across retinal layer is reported in Fig 8. In retinas of LD group, there was an increase of Iba1 positive cells particularly evident in the ONL including sub-retina compared to the Control.

**Fig 9:**
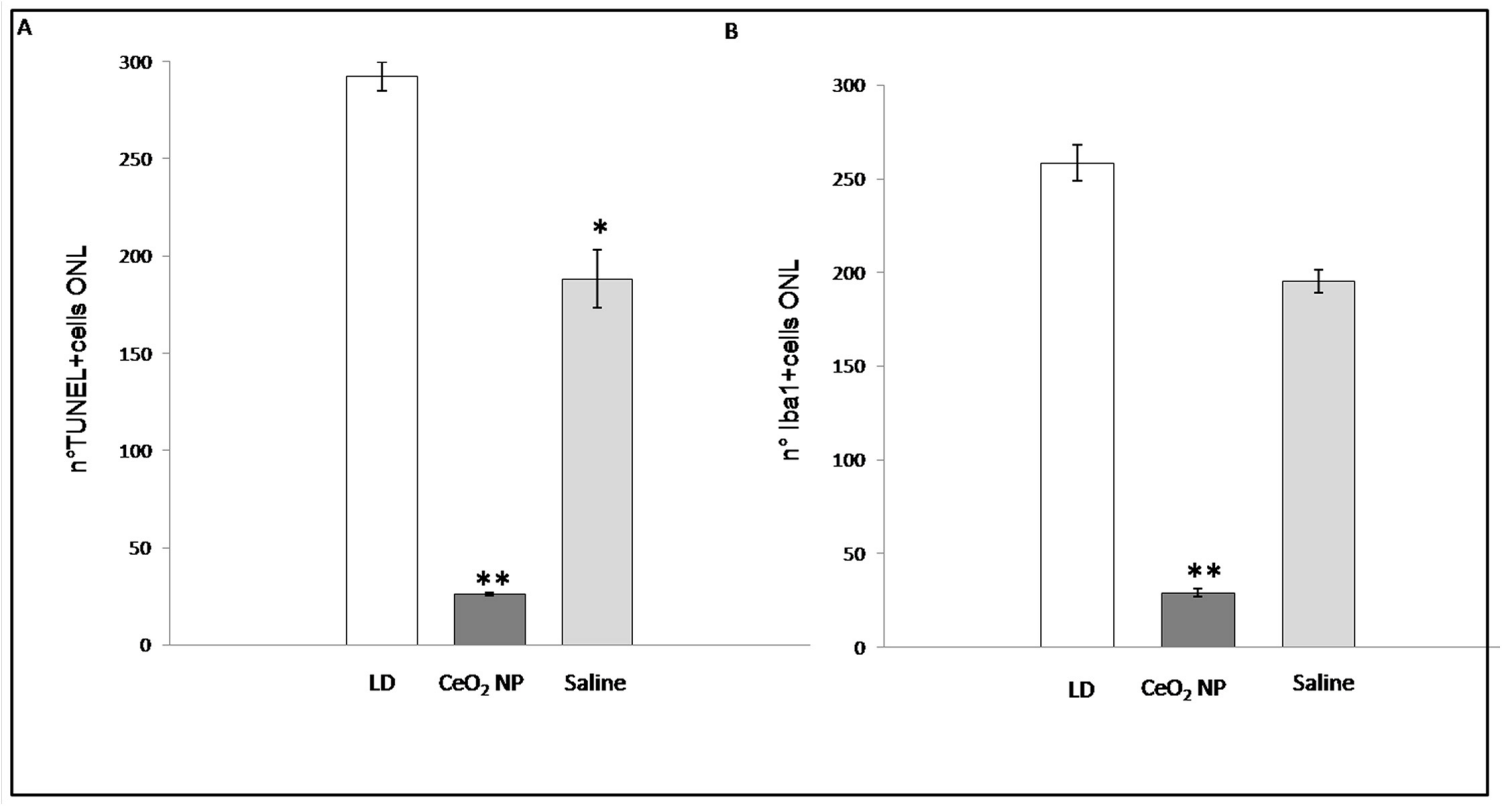
Total counting of microglia and dying neurons in ONL. Panel A: number of TUNEL-positive nuclei in the ONL in LD, CeO_2_ NP and Saline groups, one week after BCL. Panel B: number of Iba1-positive cells in ONL in LD, CeO_2_ NP and Saline groups, one week after BCL. Statistical analysis was performed, for each group versus LD. Mean ± SE is reported. *P<0.05 and **P<0.01, (Student’s t-test).

Intravitreal treatment significantly reduced Iba1-positive cells with respect to LD mainly in the ONL. The total number of dying neurons was strongly reduced in CeO_2_ NP group but it has to be noted (Fig 9A and 9B) that a significant reduction of dying neurons can be detected also in the contralateral eye (Saline), probably due to the crosstalk between the two eyes as previously suggested [17].

**Fig 10:**
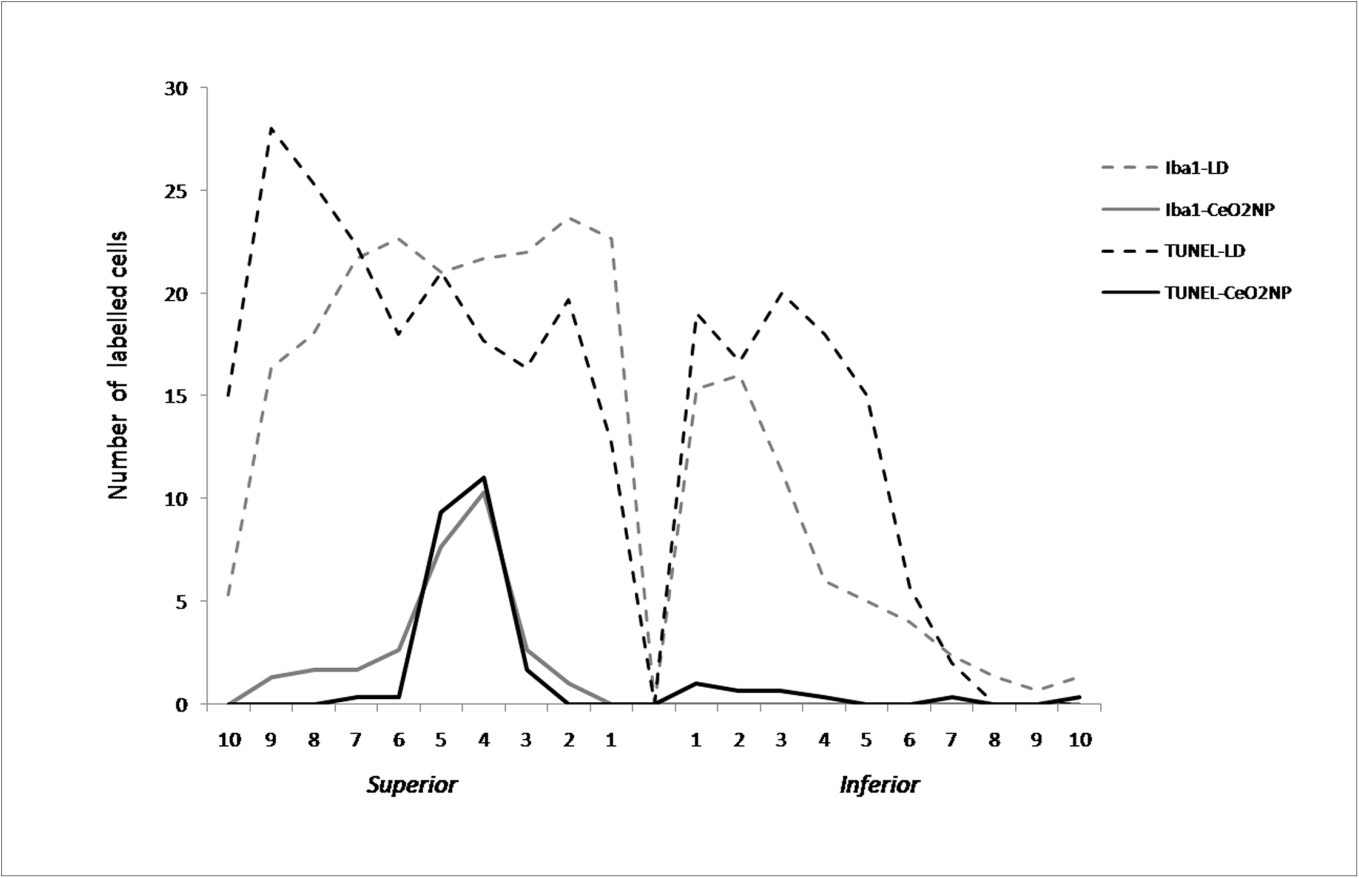
Counting of microglia and dying neurons along the entire retina. Iba1-positive cells and TUNEL-positive nuclei from superior to inferior edge of retina in CeO_2_ NP group compared to the LD group.

In Fig 10, we reported the distribution of positive TUNEL and Iba1 cells counted across the entire ONL extension from dorsal to ventral in CeO_2_ NP and LD groups. There is a clear correlation between the number of dying neurons and activated microglia; they are located in the same retinal position, which is restricted to the central part of the “hot spot” in the treated eye (CeO_2_ NP).

### Intravitreal injection of CeO_2_ Nanoparticles reduces TNF-α

TNF-α is a secreted pro-inflammatory cytokine enrolled in several cellular events, including apoptosis, cell survival, and proliferation see for ref. [18]. There are evidences suggesting its involvement in several neurological diseases including multiple sclerosis, Alzheimer’s disease and ischemia. TNF-α was found in retinas of patients as well as in animal models of retinal injury see for ref. [19].

**Fig 11:**
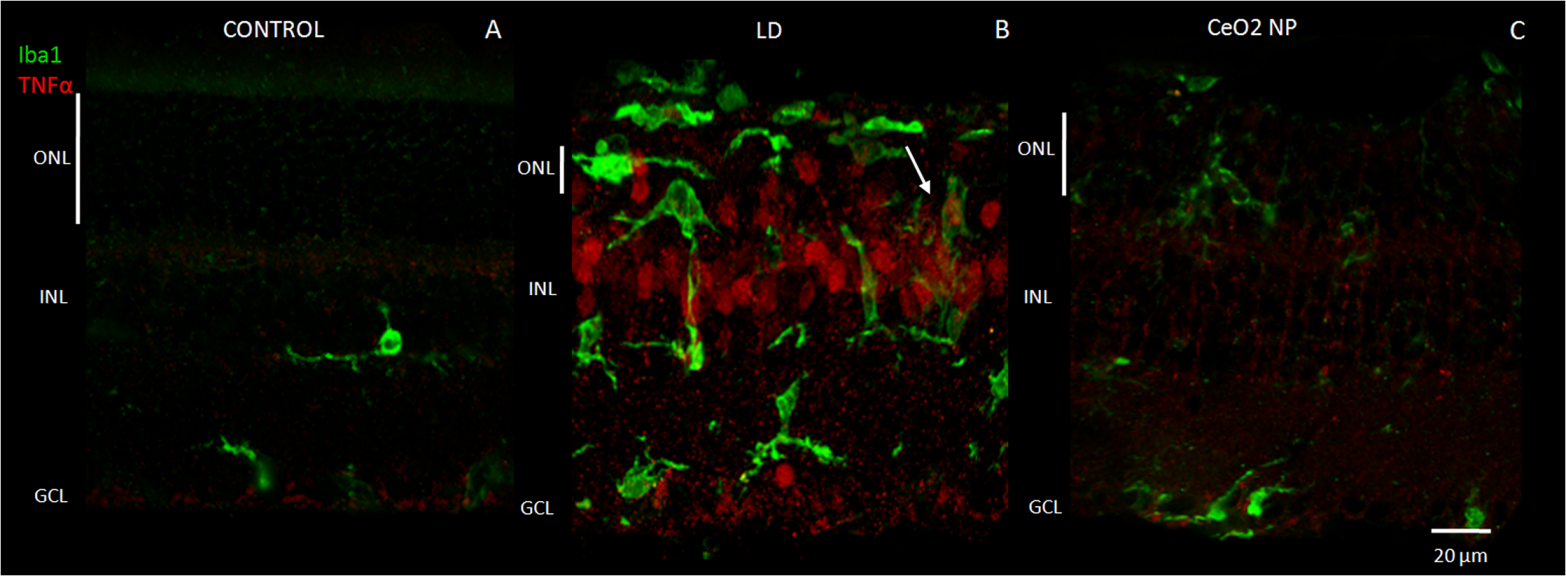
TNF-α immunolabelling in “hot spot” region. Images show the double immunolabelling for microglia (green) and TNF-α (red) in: Control (A), LD (B) and CeO_2_ NP (C). The arrow indicates the phagocitic activity of activated microglia in the ONL. Scale bar: 20 μm. ONL: outer nuclear layer, INL: inner nuclear layer, GCL: ganglion cell layer.

To show the localization of activated microglia and TNF-α in Control, CeO_2_ NP and LD groups, we reported in Fig 11 confocal images of immunolabelled retinal sections. It is interesting to note that in LD group there was a huge immune-reactivity for TNF-α especially in the inner nuclear layer (INL), into the cell bodies of Müller cells, in secondary neurons and in some dying photoreceptors in the ONL. This localization was particularly evident in the “hot spot” area and it was coupled with activated microglia as reported above (Figs 6 and 7). There was no co-localization but microglia/macrophage action seemed to be driven by TNF-α positive photoreceptors. The up-regulation of TNF-α was almost completely absent in CeO_2_ NP group; in fact, only a few extensions of Müller cells were weakly labelled. In the Control, TNF-α immune-reactivity was present only in the most inner retina at the level of limiting membrane.

### Intravitreal Injection of CeO_2_ Nanoparticles Reduces FGF2

**Fig 12:**
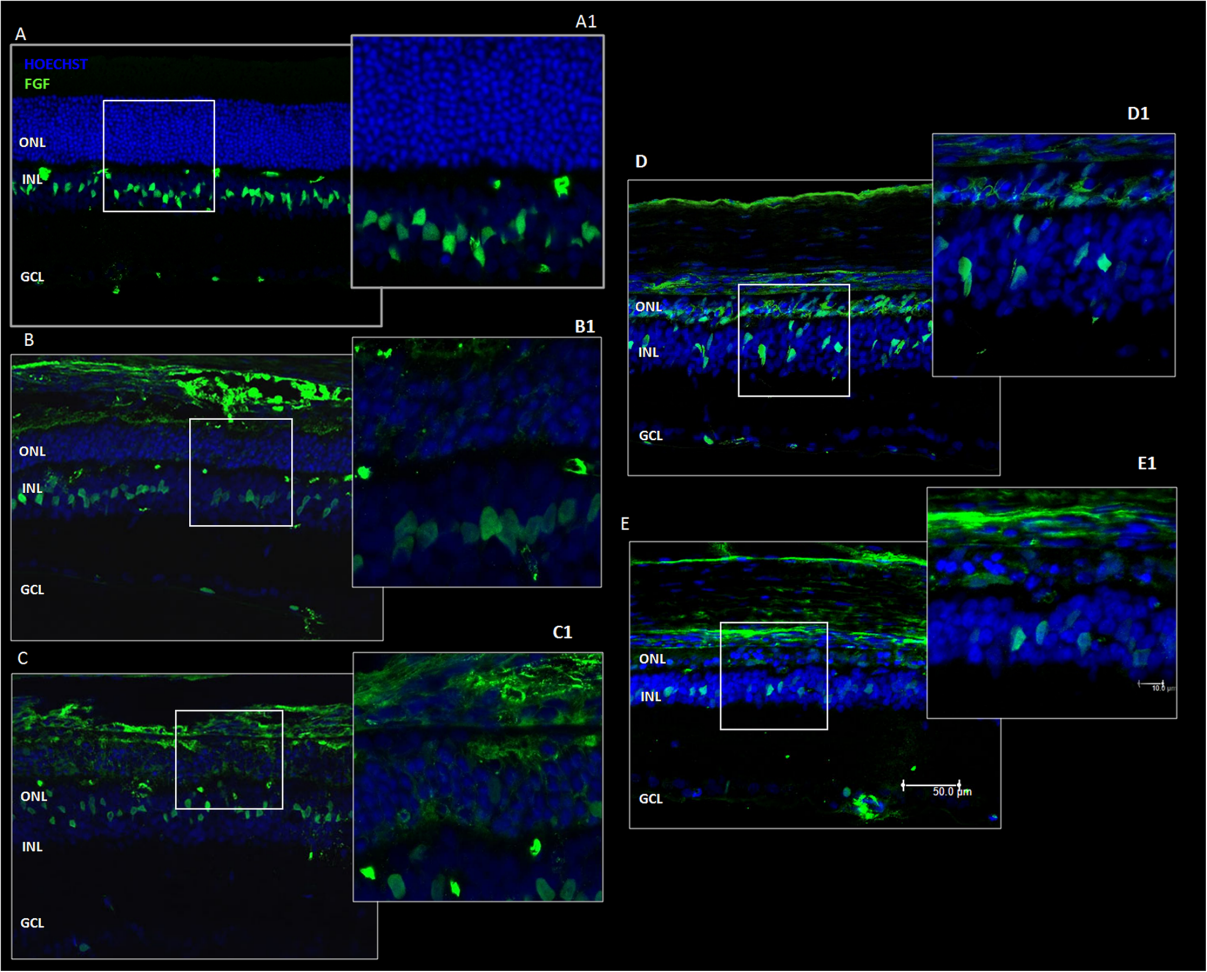
FGF2 immunolabelling in “hot spot” region. In panel A: Control, panel B: CeO_2_ NP, panel C: Saline, panel D: Vein and panel E: LD, with a scale bar of 50 μm. Panels A1-B1-C1-D1-E1: High magnification with a scale bar of 10 μm. ONL: outer nuclear layer, INL: inner nuclear layer, GCL: ganglion cell layer.

**Fig 13:**
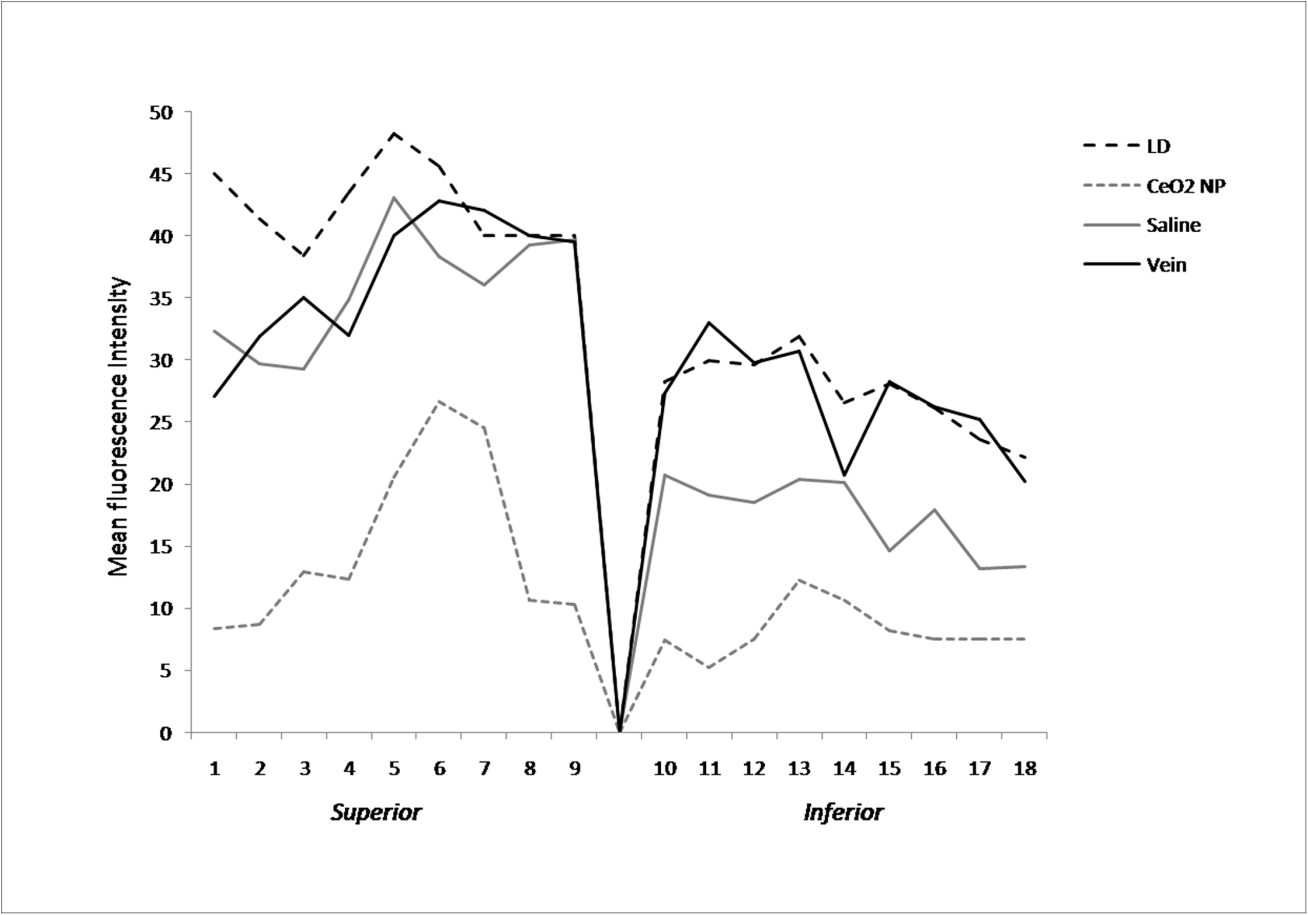
Mean fluorescence intensity analysis for FGF2. The graph represents the mean fluorescence intensity of FGF2 in the ONL from superior to inferior edge through the optic nerve. Comparison between four experimental groups: LD, CeO_2_ NP, Saline and Vein.

Previous data have shown that retinal stress induces up-regulation of trophic factors such as FGF2, which is protective for stressed photoreceptors but deleterious for visual function [16]. It is well known that in unstressed retinas, FGF2 protein is localized in Müller cells, while in retinas exposed to damaging light, it fills photoreceptors layer [6,16]. Fig 12 shows confocal images of immunolabelled retinas obtained in each experimental condition. There was no immuno-reactivity in the ONL in CeO_2_ NP group (Fig 12B) compared to other experimental groups (Fig 12C–12E). Mean fluorescence intensity analysis for FGF2 immunolabelling showed that the up-regulation of FGF2 in the ONL, after light exposure, was reduced in CeO_2_ NP group (Fig 13).

## Discussion

Our results showed that we developed and selected extremely efficient ceria nanoparticles able to reduce photoreceptor death induced by BCL, confirming previous results [8]. In addition, we provided a novel observation: the ability of ceria nanoparticles to reduce reactive response of retinal Müller cells and eventually to control microglial activation. Recently, great attention has been devoted to microglia physiology and function and there are increasing evidence of the pivotal role played by microglia in both pathogenesis and progression of neurodegenerative diseases where neuroinflammation is involved including age related macular degeneration see for ref [10,11]. It is well known that, in response to stress of different nature, there is an activation of Müller cells (reactive gliosis) well documented by increased expression of the protein GFAP [6]. Müller cells are responsible for both synthesis and release of protective factors as FGF2 and many molecules associated to a variety of functions, among the other chemokines and inflammation-associated molecules that cause microglial activation and their migration. In Control group microglia was confined in the inner layer and mainly in the two plexiform layers where they monitor synaptic activity and neurotransmitter release. The general role of microglia was very dynamic and essential in neuronal maintenance. In response to stress-injury-related signals there was an activation of microglia migration and phagocytosis. Microglia migrated in the outer retina starting from the “hot spot” region and progressively invaded the adjacent area. In CeO_2_ NP group, the “hot spot” was restricted to a smaller area, the morphology of the retina was highly preserved and microglia and apoptotic figures overlap in the same place (Fig 10) suggesting a link between neuronal death and microglia. The activation of self-protective mechanism (FGF2) is limited and visual function is well maintained see for ref. [16]. A cross talk between the two eyes was clearly demonstrated [17] after intracranial section of optic nerve. Interestingly also in this model a crosstalk was present and it is statistically significant for apoptosis (Fig 9). In LD model, oxidative stress played a relevant role. Consequently, the production of reactive oxygen species might be the first target of these strong antioxidant nanoparticles as already reported [1,8] but the pathways activated by oxidative stress appear more complex and articulated. The ability to modulate the Ce^3+^ ions concentration during the growth procedure [15] allowed us to obtain non-stoichiometric cerium oxide (CeO_2-x_) nanoparticles. In this way, CeO_2-x_, that continuously switches its oxidation state from Ce^3+^and Ce^4+^ and vice versa [8], played a critical role in its catalytic and medicine functionalities with no need of repetitive injections. In agreement with the previous considerations, our CeO_2_ nanoparticles presented a high probability to be effective as free-radical scavengers reducing the neurodegenerative processes activated by BCL. The reduction in neuroinflammation might be a consequence of reduced concentration of free-radicals, which activates a cascade of events starting from glial reactions and ending in neurodegenerative progression see for ref [2]. Our analysis showed that all these events were regulated by the presence of CeO_2_ nanoparticles: reduced activation of self protective mechanism (FGF2), reduced proapoptotic molecules (TNF-α), reduced microglia migration, preserved morphology and function. The possibility to block the negative feedback induced by degenerative events makes CeO_2_ nanoparticles an interesting treatment for neuronal diseases related to oxidative stress, nevertheless, the implications in clinical practice needs to be deeply evaluated. An interesting question is: What happens after nanoparticles have been injected in the vitreous? Previous authors [20] reported the presence of CeO_2_ nanoparticles in retinal tissue. We extended the analysis by providing the first evidence that nanoparticles are able to cross the entire retina. In fact after 24 hours from the injection they are located in the inner retina and after three weeks they are visible in the outer retina, where they remain for a long time. Nanoceria introduced intraveinously reached the retina after 24 hours but it was impossible to detect them after three weeks. In addition, our results showed that CeO_2_ nanoparticles provided with blood stream were unprotected from light damage.

## Conclusion

The results of this study concluded that CeO_2_ nanoparticles strongly reduce neuronal death and inflammatory response in a rat model of retinal neurodegeneration. In addition, retinal function is highly preserved. CeO_2_ nanoparticles injected in the vitreous remain stable in the outer layer and probably their action continues for long time. Based on these results, it is possible to consider non-stoichiometric cerium oxide nanoparticles as a convenient treatment in degenerative retinal processes, where oxidative stress plays a relevant role.
